# Development and Application of a QuEChERS-Based Liquid Chromatography Tandem Mass Spectrometry Method to Quantitate Multi-Component *Alternaria* Toxins in Jujube

**DOI:** 10.3390/toxins10100382

**Published:** 2018-09-21

**Authors:** Cheng Wang, Yingying Fan, Weizhong He, Dongqiang Hu, Aibo Wu, Wenliang Wu

**Affiliations:** 1 Beijing Key Laboratory of Biodiversity and Organic Farming, College of Resources and Environmental Sciences, China Agricultural University, Beijing 100193, China; wangcheng312@xaas.ac.cn; 2 Management of Scientific Research, Xinjiang Academy of Agricultural Sciences, Urumqi 830091, China; 3 Key Laboratory of Agro-Products Quality and Safety of Xinjiang, Laboratory of Quality and Safety Risk Assessment for Agro-Products (Urumqi), Ministry of Agriculture and Rural Affairs, Urumqi 830091, China; fyyxaas@xaas.ac.cn (Y.F.); hewei198112@xaas.ac.cn (W.H.); 4 Institute of Quality Standards & Testing Technology for Agro-Products, Xinjiang Academy of Agricultural Sciences, Urumqi 830091, China; 5 CAS Key Laboratory of Nutrition, Metabolism and Food Safety, Shanghai Institute of Nutrition and Health, Chinese Academy of Sciences, University of Chinese Academy of Sciences, Shanghai 200031, China; dqhu@sibs.ac.cn (D.H.); abwu@sibs.ac.cn (A.W.)

**Keywords:** *Alternaria* toxins, jujube, QuEChERS, UPLC-MS/MS

## Abstract

A simple, rapid and efficient methodology was developed and validated for the analysis of four *Alternaria* toxins in jujube: Tenuazonic acid, alternariol, alternariol monomethyl ether, and tentoxin. Under the optimized extraction procedure, chromatographic conditions, and instrumental parameters, the four toxins were effectively extracted via a quick, easy, cheap, effective, rugged, and safe (QuEChERS) method, and quantified by ultra-performance liquid chromatography coupled to tandem mass spectrometry (UPLC-MS/MS). Matrix-matched calibrations ranging from 0.01 to 0.5 μg mL^−1^ were conducted for the quantification due to the matrix effect. A blank jujube sample was spiked at 40, 80 and 160 μg kg^−1^, obtaining recoveries in the range of 83.5–109.6%. Limits of detection and limits of quantification were in the range of 0.14–0.26 and 0.47–0.87 μg kg^−1^, respectively. Finally, the developed method was applied for the quantification of the four toxins in 14 jujube samples, including black spot-infected and uninfected samples. Results showed that the predominant toxin detected in all the samples was tenuazonic acid, the content of which was associated with the infection level; alternariol, alternariol monomethyl ether, and tentoxin were detected in all the infected samples and some of the uninfected samples with rather low contents.

## 1. Introduction

*Alternaria* toxins are the toxic metabolites of *Alternaria* species, which are widely distributed fungi in nature, that can act as both plant pathogens and saprophytes that damage agronomic crops during their growth and post-harvest. More than 70 mycotoxins and phytotoxins have been isolated from the *Alternaria* species [1]. Among them, alternariol (AOH), alternariol monomethyl ether (AME), tenuazonic acid (TeA), tentoxin (TEN), altenuene (ALT), altertoxins I (ATX-I), and altertoxins II (ATX-II) have been reported as toxic food contaminants, widely detected in food and feedstuff infected with *Alternaria* species.

The *Alternaria* species are plant pathogens that are able to spoil cereals, fruits, and vegetables in storage or during transport, even when these are refrigerated. Considerable literature has reported the occurrence of *Alternaria* toxins in various agricultural products, such as tomatoes and tomato products [2], pomegranate fruits and juices [3], carrots [4], sunflower products [5], cereals [6], citrus-based food [7], wine [8], and dried fruits [9]. As to the analytical procedures, *Alternaria* toxins are usually extracted via liquid-liquid extraction (LLE) using an organic–water solution (acetonitrile [10], methanol [11], and ethyl acetate [12]) mixed with acetic acid, which is preferable for the extraction of TeA, an acidic compound [10]. However, this procedure is tedious and the large number of required toxic organic solvents is time- and resource-consuming. Given its low consumption of highly toxic organic solvents and the clean-up effort required, solid-phase extraction (SPE) was developed to determine *Alternaria* toxins using hydrophilic–lipophilic balance (HLB) [7,13], octadecyl silane (C18) [14,15], aminopropyl [16], and polystyrene-divinylbenzene copolymer (PS-DVB) SPE cartridge [17]. Nevertheless, SPE procedures include activation, elution, and evaporation steps, which is labor-intensive to some extent. To avoid these drawbacks, some efficient and miniaturized sample pretreatment methods were developed by a number of analysts. Fan et al. [18] proposed a novel pretreatment method, ionic liquid modified countercurrent chromatography to extract AOH, AME, and TeA from wine and juice. Font et al. [19] developed the dispersive liquid-liquid microextraction (DLLME) coupled to gas chromatography-mass spectrometry (GC-MS) for the monitoring of AOH, AME, and ALT in tomatoes. From a practical point of view, the application of these novel methods is limited to the sample matrix and types of *Alternaria* toxins.

In 2003, Anastassiades [20] first introduced the QuEChERS (quick, easy, cheap, effective, rugged, and safe) method for pesticide analysis in fruits and vegetables, in which sorbents were added into sample solutions directly without the use of SPE columns. Because of its simplicity, efficacy, and adaptability to multiresidue analysis, QuEChERS was successively adopted as the standard pretreatment method in Association of Official Analytical Chemists (AOAC) Official Method 2007.01 [21] and British Standard and European Standard Norme (BS EN) 15662:2008 [22]. The applications of this method have expanded to pesticides, veterinary drugs, endocrine disrupting chemicals, and mycotoxins in a wide variety of matrices [23], which also include the detection of *Alternaria* toxins in tomato products [24], vegetable juices [25], pomegranate fruits and juices [3], cereals [26], cereal-based foodstuffs [6], citrus [27], barley [28,29], soya beans [30], and animal feed [31]. However, the extraction of *Alternaria* toxins from jujube has not been studied to date.

Because of the unique composition of sugar, water, and the nutrient content during growth and post-harvest, jujube is susceptible to infection by fungi and even encourages fungal growth [32]. Therefore, the aim of this study was to develop a novel analysis method using QuEChERS extraction coupled with ultra-performance liquid chromatography coupled to tandem mass spectrometry (UPLC-MS/MS) detection for the analysis of AOH, AME, TeA, and TEN in jujube. This methodology was then applied to determine the presence of the *Alternaria* toxins in black spot-infected and -uninfected jujube samples collected from South Xinjiang, China. This is the first report related to the analysis of *Alternaria* toxins in jujube. 

## 2. Results and Discussion

### 2.1. Optimization of Tandem Mass Spectrometry (MS/MS) Instrumental Detection

In this study, the MS/MS conditions of the *Alternaria* toxins were optimized via infusing the individual standard at 0.5 μg mL^−1^. Positive and negative electrospray ionization (ESI) modes were tested for the analytes, and as previously reported in the literature [33,34], ESI in positive mode (ESI+) was selected as it produced the best results in terms of sensitivity. Other parameters were also optimized for each *Alternaria* toxin and the results are shown in Table 1.

### 2.2. Optimization of Chromatographic Conditions

To obtain the optimized chromatographic conditions, mixtures of standard solutions of *Alternaria* toxins in acetonitrile and in the extract of jujube after QuEChERS were used. On the basis of a previous paper [8], the gradient conditions were slightly modified to achieve more symmetrical and sharper peaks with a shorter retention time. Different acids (formic and acetic acid) in the mobile phases were tested to improve the ionization in ESI (+) and results showed that formic acid provided better results. Hence, the double mobile phases with 0.1% formic acid were selected. In addition, different columns (Waters Acquity UPLC High Strength Silica (HSS) T3 column and Ethylene Bridged Hybrid (BEH) C18 column) were also evaluated. Results showed that the BEH C18 column (Figure S1a) could advance the retention time for the four analytes, but its separation was not good compared to that of the HSS T3 column (Figure S1b). Thus, in the following experiment, the HSS T3 column was applied. 

### 2.3. Optimization of QuEChERS

In order to create a quick and effective extraction method, QuEChERS was selected as it has been previously used to extract *Alternaria* toxins from pomegranate fruits and juices [3], cereal-based foodstuffs [6], tomato products [24], and citrus [27], but it has not been tested for jujube. Thus, the aim of this study was to investigate if QuEChERS is suitable for extracting *Alternaria* toxins (AOH, AME, TeA, and TEN) from jujube. In what follows, the ratio of sample/water, type and volume of extraction solvent, amount of salts, and type of clean-up agent are optimized, whereas the other parameters including equilibration time (10 min), extraction time (5 min), and centrifugation time (5 min) were selected based on the literature [3] and our previous experience. 

#### 2.3.1. Selection of Raw Sample Treatment Method

Xinjiang jujube has a high content of sugar because the temperature in which it grows is significantly different between day and night. It is difficult to stir dried jujube like other fruit and vegetables. Thus, in this study, we removed the date stone once we obtained the fresh jujube. Different treatment methods (directly homogenized via an ultra-turrax and freeze-drying grinding under liquid nitrogen) were optimized. Though the obtained freeze-dried powder has a higher contact area, it easily clusters when exposed to air or water, which complicates homogenization through shaking by hand or apparatus. Thus, we chose the direct-homogenization method. 

**Figure 1:**
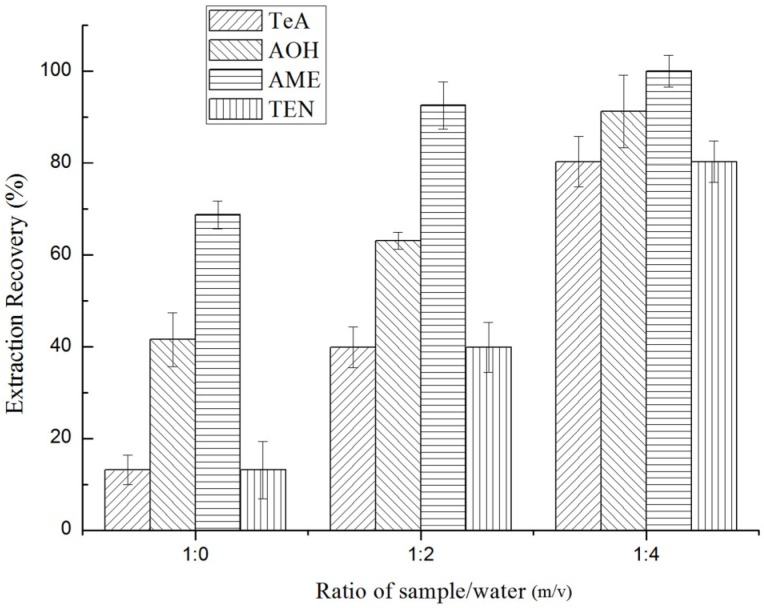
Effect of the ratio of sample/water (*m/v*) on the extraction recoveries (ERs%) of four *Alternaria* toxins: tenuazonic acid (TeA), alternariol (AOH), alternariol monomethyl ether (AME), and tentoxin (TEN).

Initially, QuEChERS extraction was targeted at the matrix of water-rich vegetables and fruits. However, when the samples have a water content below 80%, it is recommended to add additional water. Therefore, water was added to jujube samples, and different sample/water ratios from 1:0 to 1:4 were investigated. As seen in Figure 1, with the increase in the ratio, the extraction recoveries (ERs%) of all *Alternaria* toxins increased. This could be explained by the high content of water helping to swell the matrix and weaken the interactions of analytes with matrix components [35], which led to an efficient extraction. Thus, 2.5 g jujube and 10 mL water were selected to conduct the experiment.

#### 2.3.2. Selection of Extraction Solvent

**Figure 2:**
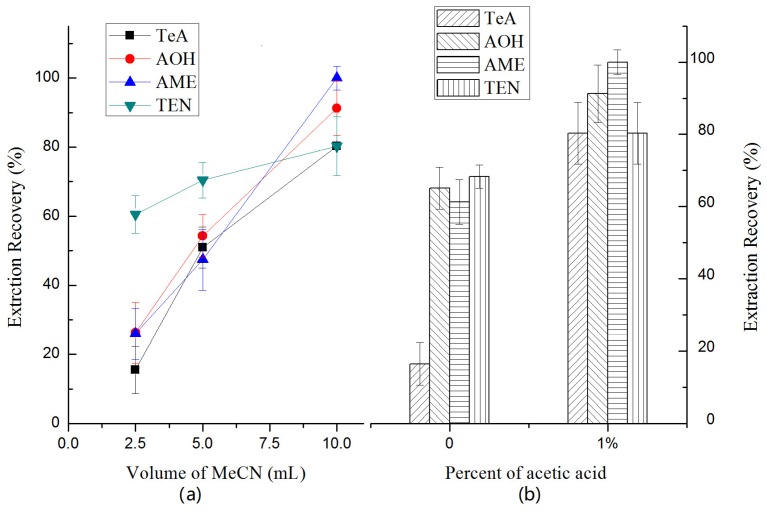
Effect of (**a**) the volume of MeCN and (**b**) percent of acetic acid on the ERs% of four *Alternaria* toxins: TeA, AOH, AME and TEN.

The main factor used to choose the extraction solvent was its miscibility with analytes. Traditional organic solvents such as acetonitrile, ethanol, and methanol, were tested using 1 g of NaCl and 4 g of MgSO_4_. As the phase separation in the salting-out step was impossible with methanol and ethanol, acetonitrile was the chosen alternative. Different acetonitrile volumes (2.5, 5, and 10 mL) were tested to study its influence on the ERs% of the analytes. Figure 2a shows that the ERs% of each *Alternaria* toxin increased gradually as the volume increased. However, as larger volumes might have induced the enrichment factor, we did not investigate larger volumes. Thus, 10 mL acetonitrile was adopted in the following study. Acidification of acetonitrile with 1% acetic acid was also tested. As shown in Figure 2b, the acid improved the ERs% for all *Alternaria* toxins, especially for TeA. 

#### 2.3.3. Selection of Salt Addition

**Figure 3:**
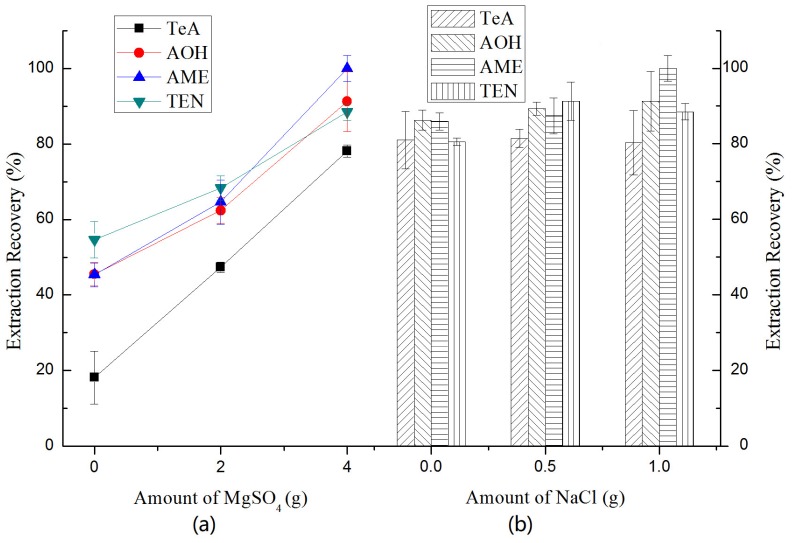
Effect of (**a**) the amount of MgSO_4_ and (**b**) NaCl on the extraction recoveries (ERs%) of four *Alternaria* toxins: TeA, AOH, AME and TEN.

In general, the addition of salt during QuEChERS extraction could separate water from the organic solvent based on the salting-out effect, and these salts generally include anhydrous magnesium sulfate (MgSO_4_), sodium chloride (NaCl), anhydrous sodium citrate (Na_3_Cit), and sodium acetate (NaOAc). In this study, we selected anhydrous MgSO_4_ and NaCl as the salting-out agents and different amounts were investigated individually. From Figure 3, we see that the ERs% for all the *Alternaria* toxins increased as the amount of anhydrous MgSO_4_ increased from 0 to 4 g and was significantly unchanged as the amount of NaCl increased from 0 to 1 g. Thus, we concluded that the classical combination of 4 g of anhydrous MgSO_4_ and 1 g NaCl in most studies was also adopted in this study.

#### 2.3.4. Selection of Clean-Up Agent

Generally, some amounts of interfering compounds in the matrix could also be co-extracted and injected into the UPLC-MS/MS, which could reduce the column lifetime and influence the detection [36]. Thus, it was necessary to add a clean-up procedure after the extraction. Traditional clean-up agents, such as primary secondary amine (PSA), Florisil, octadecyl silane (C18), and graphitized carbon black (GCB), were tested in this study. As shown in Figure S2, the ERs% for all the *Alternaria* toxins reduced when the clean-up agent was added, especially Florisil and GCB, which might have adsorbed the *Alternaria* toxins. Therefore, no clean-up agent was used in the following experiment.

### 2.4. Method Validation

Components in the matrix can influence the ionization when ESI is used, and the stable isotope dilution assay is the best method available to compensate for the matrix effect [37]. However, most of the isotope internal standards are usually not available and expensive. In this study, the standard curves were established by plotting five concentration levels in the range of 0.01 to 0.5 μg mL^−1^ against peak areas in pure acetonitrile and in a blank jujube sample. According to Frenich [36], if the slope ratio of the matrix/solvent for each analyte was in the range of 0.8–1.2, it indicated that there is no matrix effect, otherwise a strong matrix effect exists. The slope ratios shown in Table 2 indicated a strong matrix effect for each *Alternaria* toxin. Thus, matrix-matched calibration standard curves were adopted to quantify *Alternaria* toxins in jujube samples. Calibration coefficients (R^2^) ranged from 0.9866 to 0.9997. Limit of Detection (LOD) and Limit of Quantitation (LOQ) varied in the range of 0.14–0.26 μg kg^−1^ and 0.47–0.87 μg kg^−1^, respectively, which were comparable with those obtained by liquid liquid extraction-liquid chromatography (LLE-LC)-MS/MS [5,10], solid phase extraction-liquid chromatography (SPE-LC)-MS/MS [2,8] and QuEChERS-LC-MS/MS [25,27]. Recovery experiments were conducted via spiking three different concentrations (40, 80, and 160 μg kg^−1^) of *Alternaria* toxins for a jujube sample (S-7), which had the lowest *Alternaria* toxins content. Satisfactory recoveries of all the toxins were obtained in the range of 83.5–109.6% (Relative standard deviation, RSD ≤ 6.5%). These results described above demonstrated that the developed method was reliable for the analysis of *Alternaria* toxins in jujube. Figure S3 shows the LC-MS/MS chromatograms of the blank and spiked (80 μg kg^−1^) jujube sample (S-7).

### 2.5. Sample Analysis

To check the applicability of the developed method, 14 jujube samples including black spot-infected and -uninfected jujubes from seven jujube yards in South Xinjiang were analyzed. From Table 3, we can see that TeA was found in lower levels in all the samples, and the amounts were associated with the infection levels: Uninfected jujube (<LOQ ~1.0933 μg g^−1^) <infected jujube (0.1373–8.2375 μg g^−1^), and AOH (0–0.0228 μg g^−1^), AME (0–0.0692 μg g^−1^), and TEN (<LOQ ~0.0247 μg g^−1^). Except for uninfected jujubes, in the other infected samples the four *Alternaria* toxins were all detected. The distinction of *Alternaria* toxins detected in black spot-infected jujubes among the seven yards was caused by the type of *Alternaria* species [38] and environmental factors [39].

## 3. Conclusions

A novel method based on QuEChERS extraction coupled to UPLC-MS/MS detection was proposed for the first time for the detection of four *Alternaria* toxins in jujube. The extraction procedure is simple and no clean-up procedure is needed. Furthermore, the use of UPLC-MS/MS provides a fast and reliable detection of *Alternaria* toxins. Under the optimized chromatographic, MS/MS detection and sample pretreatment conditions, satisfactory method validation parameters including linearity, precision, recovery, LOD, and LOQ, were obtained. Additionally, matrix-matched calibration was successfully used to compensate for the strong matrix effect. Finally, this method was applied to 14 jujube samples including infected and uninfected samples collected from South Xinjiang, China. Results showed that the *Alternaria* toxins exist in jujube samples, especially jujubes infected by black spot.

## 4. Materials and Methods

### 4.1. Reagents

AOH, AME, TeA, and TEN were provided by Romer Labs Division Holding GmbH (Getzersdorf, Austria). Methanol and acetonitrile at HPLC grade were obtained from Thermo Fisher Scientific (Waltham, MA, USA). Florisil, PSA, C18, GCB with diameters from 20 to 30 nm were also supplied by Thermo Fisher Scientific (Waltham, MA, USA). Sodium chloride and anhydrous magnesium sulfate were purchased from Urumqi Chemical Reagent (Urumqi, China). 

Individual standard solutions of each *Alternaria* toxin were prepared at ca. 100 μg mL^−1^ in acetonitrile and stored at −20 °C. The mixture working standard solution (1 μg mL^−1^) was prepared by diluting the individual standard solutions and stored at 4 °C. 

### 4.2. Instrument

The detection of *Alternaria* toxins was carried out on a Waters Acquity UPLC- tandem quadrupole (TQD) mass spectrometer (Waters, Milford, MA, USA), which contained an Acquity UPLC HSS T3 (1.8 μm, 2.1 × 100 mm) column for separation. Column temperature was set at 40 °C. The mobile phase was comprised of acetonitrile containing 0.1% formic acid as eluent A and ultrapure water containing 0.1% formic acid as eluent B. A gradient elution was applied as follows: 5% A was initially used and linearly increased to 80% within 2.5 min, then to 90% within 2 min, then maintained for 1.5 min, after which, column re-equilibration took place, leading to a total run time of 6 min. The flow rate was set at 0.3 mL min^−1^.

The MS/MS analysis was operated in the positive mode at a capillary voltage of 3.0 kV, a desolvation temperature of 350 °C, a source block temperature of 125 °C, a desolvation gas of 800 L h^−1^, and a cone nitrogen gas flow of 50 L h^−1^. The collision gas was argon with a pressure of 4 × 10^−3^ mbar. The multiple reaction monitoring (MRM) transitions and the applied parameters are summarized in Table 1.

Samples were weighed by a XSE204 balance (Mettler-Toledo, Greinfesee, Switzerland), homogenized via a T13 basic ultra-turrax (IKA, Staufen, German), mixed with a MS3 vortex mixer (IKA, Staufen, German) and shaken with an automatic horizontal shaker (Hanuo Instruments, Shanghai, China). Centrifugation was performed with a sorvall biofuge stratos system (Thermo Fisher Scientific, Waltham, MA, USA). Water was purified using a PALL Cascada III.I system (Pall Corporation, New York, NY, USA).

### 4.3. Samples

Fourteen samples (numbered from S-1 to S-14) including black spot-infected and -uninfected jujube from seven jujube yards in South Xinjiang were analyzed. The date stones were removed from the samples in advance and then directly homogenized before stored at −14 °C until the moment of analysis.

### 4.4. Sample Treatment

For the extraction of *Alternaria* toxins, a 2.5 g homogenized sample was spiked with the mixture standards at concentrations of 1 μg mL^−1^, vortexed for 20 s, and then placed in the dark for 10 min. Next, 10 mL water and 10 mL acetonitrile containing 1% acetic acid were added successively, and then shaken with an automatic horizontal shaker at 2500 rpm for 5 min to fully disperse the sample. Subsequently, 4 g of anhydrous MgSO_4_ and 1 g of NaCl were immediately added and then shaken in the tube to prevent agglomeration of the salts. After centrifugation at 5000× *g* for 5 min, the supernatant layer was evaporated to near dryness (about 1 mL residue left) under a nitrogen stream at 40 °C. Finally, 1 mL of the combined solution (acetonitrile/methanol/formic acid, 70:29:1, v/v) was added into the residue, vortexed, filtered through a 0.22 μm nylon filter and injected into the UPLC–MS/MS system.

Additionally, a clean-up step was conducted in the optimization procedure. After the above centrifugation step, the supernatant layer (about 8 mL) was transferred into a 15 mL-centrifuge tube containing 1.2× *g* anhydrous MgSO_4_ and 0.4 g PSA (or C18, Florisil and GCB). After shaking (3 min) and centrifugation (5 min at 5000 rpm), 1 mL of the supernatant was taken, filtered through a 0.22 μm nylon filter and injected into the UPLC–MS/MS system.

## Figures and Tables

**Table 1:** Optimized tandem mass spectrometry (MS/MS) instrumental parameters for *Alternaria* toxins.

Analyte	Precursor Ion (*m/z*)	Molecular Ion	Cone Voltage (V)	Production (*m/z*)	Collision Energy (eV)	Retention Time (min)
**Tenuazonic acid (TeA)**	198.92	[M + H] +	35	124.8 ^1^ 152.9	15 14	3.54
**Alternariol (AOH)**	258.9	[M + H] +	34	184.9 213.0	25 27	3.52
**Alternariol monomethyl ether (AME)**	272.9	[M + H] +	35	257.9 226.8	26 27	4.12
**Tentoxin (TEN)**	415.0	[M + H] +	36	199.0 170.9	14 20	3.54

^1^ The underlined product ion was the most abundant and thus used for quantification purposes.

**Table 2:** Analytical and statistical parameters of the proposed method for the determination of *Alternaria* toxins.

Analyte	TeA	AOH	AME	TEN
Linear range (μg mL^−1^)	0.01–1	0.01–1	0.01–1	0.01–1
Calibration curves in pure solvent				
Equation of the curve	y = 9229.1x − 323.72	y = 19,285x + 863.15	y = 23,975x + 983	y = 221,548x + 2969.8
Correlation coefficient (R^2^)	0.9996	0.9893	0.9855	0.9922
Calibration curves in a blank jujube sample				
Equation of the curve	y = 2489.1x − 54.376	y = 12,613x − 471.32	y = 15,189x − 371	y = 73,096x + 495.47
Correlation coefficient (R^2^)	0.9897	0.9997	0.9866	0.9980
Slope ratio	0.2697	0.6540	0.6335	0.3299
Limit of Detection (LOD) (μg kg^−1^)	0.14	0.26	0.22	0.20
Limit of Quantitation (LOQ) (μg kg^−1^)	0.47	0.87	0.72	0.66
Intra-day precision (RSD%^1^, *n* = 3) ^2^	0.1	2.8	1.1	0.8
Inter-day precision (RSD%, *n* = 3) ^2^	2.4	3.0	5.0	1.9
Average recovery ± RSD% (*n* = 3) for red jujube				
Concentration (μg kg^−1^)				
160	91.0 ± 1.4	91.3 ± 0.9	109.6 ± 0.4	89.3 ± 3.6
80	87.1 ± 4.4	86.0 ± 2.9	93.0 ± 4.8	86.5 ± 3.3
40	83.5 ± 6.5	84.8 ± 4.2	88.6 ± 2.4	85.2 ± 2.7

^1^ Relative standard deviation; ^2^ Spiked at 80 μg kg^−1^.

**Table 3:** *Alternaria* toxins content (μg g^−1^) in different jujube samples.

No.	Sample	Location	TeA	AOH	AME	TEN
S-1	Uninfected jujube	Pishan County, Hotan Prefecture	1.0933 (0.62) ^1^	<LOQ ^2^	<LOQ	0.0104 (3.05)
S-2	Infected jujube		8.2735 (3.62)	0.0228 (0.69)	0.0692 (1.85)	0.0074 (3.42)
S-3	Uninfected jujube	The 8th Company, the10th Regiment, Alar City	0.1817 (0.33)	<LOQ	nd ^3^	0.0078 (3.21)
S-4	Infected jujube		3.3141 (2.58)	<LOQ	<LOQ	0.0064 (4.63)
S-5	Uninfected jujube	The 5th Company, the 224th Regiment, Hotan Prefecture	0.2944 (4.39)	<LOQ	<LOQ	0.0029 (3.78)
S-6	Infected jujube		0.8390 (0.55)	<LOQ	<LOQ	0.0095 (3.41)
S-7	Uninfected jujube	The 20th Company, the 50th Regiment, Alar City	<LOQ	nd	<LOQ	<LOQ
S-8	Infected jujube		0.1879 (1.04)	nd	nd	<LOQ
S-9	Uninfected jujube	The 5th Company, the 14th Regiment, Alar City	0.1506 (5.04)	0.0012 (8.33)	0.0046 (2.38)	0.0247 (0.55)
S-10	Infected jujube		0.8506 (1.62)	<LOQ	<LOQ	0.0058 (1.72)
S-11	Uninfected jujube	The 12th Company, the 224th Regiment, Hotan Prefecture	0.0964 (8.66)	<LOQ	<LOQ	0.0086 (1.64)
S-12	Infected jujube		0.1373 (0.11)	0.0015 (6.67)	0.0011 (9.09)	0.0072 (1.55)
S-13	Uninfected jujube	The 8th Company, the 224th Regiment, Hotan Prefecture	0.4179 (0.42)	<LOQ	<LOQ	0.0053 (2.63)
S-14	Infected jujube		5.3881 (5.49)	<LOQ	<LOQ	0.0070 (1.43)

^1^ Mean (RSD%), *n* = 3; ^2^ below the quantification limit; ^3^ not detected.

## Supplementary Materials

The following are available online at http://www.mdpi.com/2072-6651/10/10/382/s1, Figure S1: Optimization of chromatographic column for the four analytes. (a) Waters Acquity Ultra-Performance Liquid Chromatography (UPLC) Ethylene Bridged Hybrid (BEH) C18 column (1.8 μm, 2.1 × 100 mm); (b) Waters Acquity UPLC High Strength Silica (HSS) T3 column (1.8 μm, 2.1 × 100 mm), Figure S2: Effect of the type of clean-up agent on the extraction recoveries (ERs%) of four *Alternaria* toxins: tenuazonic acid (TeA), alternariol (AOH), alternariol monomethyl ether (AME), and tentoxin (TEN), Figure S3: The UPLC- tandem mass spectrometry (MS/MS) chromatograms of the blank (a) and spiked (b) (80 μg kg^−1^) jujube sample (S-7).
