# Supplementary material for: Development and Application of a QuEChERS-Based Liquid Chromatography Tandem Mass Spectrometry Method to Quantitate Multi-Component Alternaria Toxins in Jujube
Source: Toxins (Basel). 2018 Sep 21;10(10):382. doi: 10.3390/toxins10100382 (PMC6220753; doi:10.3390/toxins10100382)
Supplement: Supplementary file 1 [file toxins-10-00382-s001.pdf]

# Supplementary Materials: Development and Application of a QuEChERS-Based Liquid Chromatography Tandem Mass Spectrometry Method to Quantitate Multi-Component *Alternaria* Toxins in Jujube

**Cheng Wang, Yingying Fan, Weizhong He, Dongqiang Hu, Aibo Wu and Wenliang Wu**

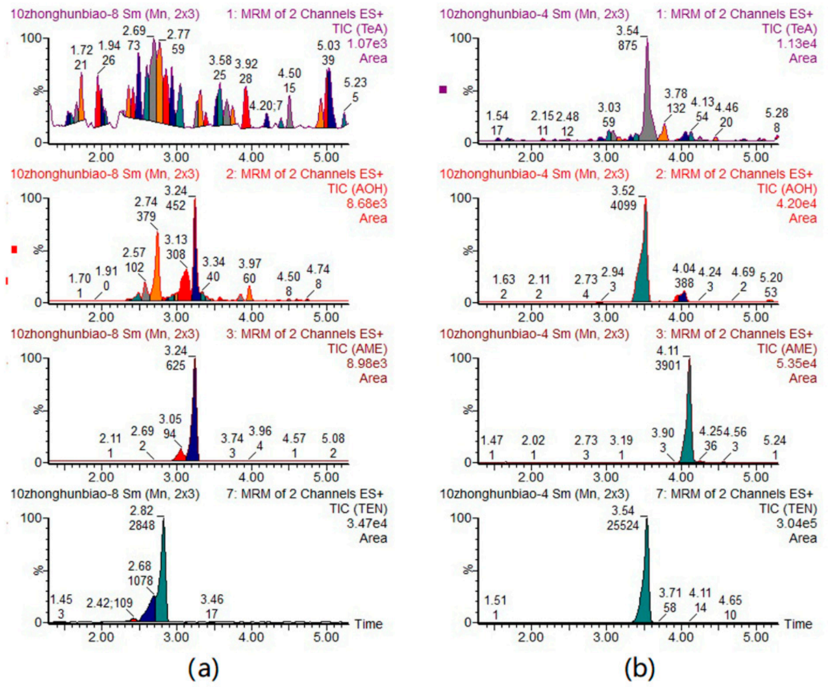


**Figure S1.** Optimization of chromatographic column for the four analytes. (**a**) Waters Acquity Ultra-Performance Liquid Chromatography (UPLC) Ethylene Bridged Hybrid (BEH) C18 column (1.8 µm, 2.1 × 100 mm); (**b**) Waters Acquity UPLC High Strength Silica (HSS) T3 column (1.8 µm, 2.1 × 100 mm).

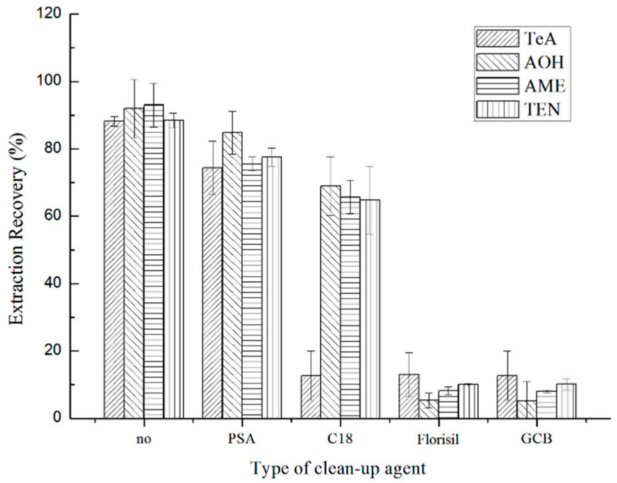

**Figure S2.** Effect of the type of clean-up agent on the extraction recoveries (ERs%) of four *Alternaria* toxins: tenuazonic acid (TeA), alternariol (AOH), alternariol monomethyl ether (AME), and tentoxin (TEN).

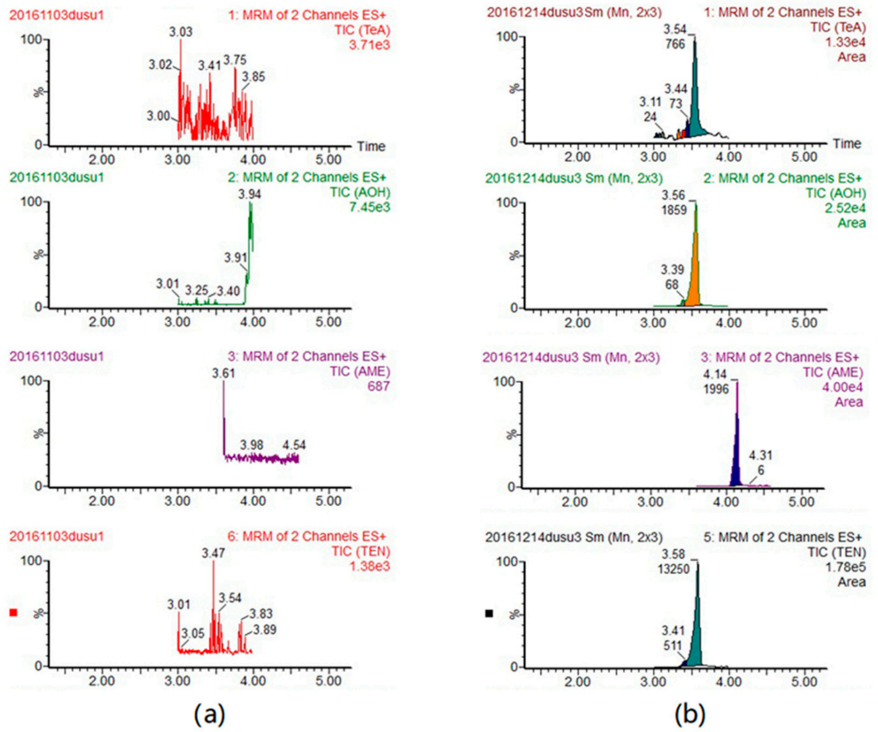


**Figure S3.** The UPLC-tandem mass spectrometry (MS/MS) chromatograms of the blank (**a**) and spiked (**b**) (80 μg $kg^{-1}$) jujube sample (S-7).
